# Trends in publication and levels of social determinants of health reporting in *Journal of Clinical and Translational Science* from 2017 to 2023

**DOI:** 10.1017/cts.2024.508

**Published:** 2024-04-02

**Authors:** Yulia A. Levites Strekalova, Xiangren Wang, Orlando Sanchez, Sara Midence

**Affiliations:** 1 Department of Health Services Research, Management and Policy, College of Public Health and Health Professions, University of Florida, Gainesville, FL, USA; 2 Clinical and Translational Science Institute, University of Florida, Gainesville, FL, USA

**Keywords:** Clinical and translational research, phenx toolkit, social determinants of health, scoping review, translational science

## Abstract

Social determinants of health affect clinical and translational research processes and outcomes but remain underreported in empirical studies. This scoping review examined the rate and types of social determinants of health (SDoH) variables included in the JCTS translational research studies published between 2017 and 2023 and included 129 studies. Most papers (91.7%) reported at least one SDoH variable with age, race and ethnicity, and sex included most often. Future studies to inform the role of SDoH data in translational research and science are recommended, and a draft SDoH data checklist is provided.

## Introduction

The inclusion of social determinants of health (SDoH) as standard data elements in research promises to improve the understanding of study participants’ individual and social circumstances and the effects of SDoH on research and health outcomes.

The role of SDoH has been extensively documented and linked to both health [1,2] and research outcomes [1,3,4]. The thorough recording of SDoH contextualizes health data, fostering targeted interventions to mitigate their adverse effects and providing valuable insights for developing personalized interventions for diverse patient populations. There is a wide-reaching consensus on the importance of capturing the SDoH systematically and rigorously. Research increasingly demonstrates how SDoH contributes to disparities observed across various demographic groups [5–8], and a growing body of evidence suggests that SDoH influence individuals' engagement in research activities [3,9,10] and can significantly affect clinical research outcomes [11,12]. Initial research has begun to establish the link between social risk factors and the results of clinical trials, suggesting that these factors may compromise trial efficacy [13,14]. However, the evidence for these assertions remains limited primarily due to the inconsistent capture and reporting of SDoH in research studies [11,15–17] Furthermore, scoping reviews have consistently indicated a significant gap in reporting social determinants, such as race, ethnicity, and demographic characteristics within the clinical and translational research literature [3,9,16,18].

## Consensus measures of the social determinants of health

In response to the need for standardized SDoH data collection, the National Institute on Minority Health and Health Disparities (NIMHD) led an effort to data capture protocols for research studies. The NIMHD convened two task forces “to improve the quality and consistency of data acquisition” across research studies [19]. The task forces vetted and selected core and supplementary instruments for inclusion in the “Consensus Measures for Phenotypes and eXposures” (PhenX) toolkit. The PhenX SDoH Core collection was designed to normalize SDoH documentation, improve research data consistency, facilitate data combination from different studies, and promote comparable SDoH data adoption across studies [20]. Introduced in May 2020, it encapsulates data capture protocols, offering assessments at both individual and social levels. It covers various SDoH domains, including environmental exposures, sociocultural community context, economic resources, employment status, food environment, health literacy, and access to healthcare.

## Study objective

The present study aimed to identify and examine the rate and types of SDoH variables included in the empirical studies published in the *Journal of Clinical and Translational Science* from its first volume published in 2017 to December 2023. SDoH domains, as defined by the NIH-developed PhenX Core SDoH toolkit, were the focus of the analysis. Specifically, this review addressed the following research questions:

RQ1: What types of SDoH variables are reported in the empirical papers published in the JCTS?

RQ2: What are the annual trends in reporting the SDoH variables in the empirical papers published in the JCTS?

## Methods

### Study design

This study conducted a systematic scoping review and followed the guidelines by the Joanna Briggs Institute [21,22] to conduct the review of the literature and develop a Preferred Reporting Items for Systematic Reviews and Meta-Analyses (PRISMA-ScR) flow chart (Appendix 1) and checklist (Appendix 2). The protocol was not registered due to the scoping nature.

### Information sources and search strategy

All papers published in the *Journal of Clinical and Translational Science* were searched using the PubMed database and included in the title and abstract screening.

### Inclusion and exclusion criteria

Studies were screened and included in the review if they (1) used primary or secondary qualitative or quantitative data or (2) reported on the recruitment or research engagement efforts that involved feedback or participation of the health consumer, community, or patient advocacy groups. After reviewing the titles and abstracts, studies focused on workforce development initiatives and training studies were excluded. Educational projects are an essential type of human participant study, but their outcomes target training and professional competency rather than health outcomes. Further exclusions were made for commentaries, policy analyses, reviews, animal studies, conceptual papers, and papers focusing on inter- or intra-institutional workflow and network development.

### Screening and data charting process

Articles were searched on PubMed by the first author (YLS), and search results were uploaded into the Covidence online platform for review management. Two authors (YLS and OS) screened the title and abstract of each paper. Screening decisions and conflicts were discussed and noted in study settings. Data from the papers included in the final review were extracted using Excel. During the extraction, the Methods and Results sections of included papers were read to identify demographic and sociographic variables aligned with the 16 domains of the PhenX Core SDoH toolkit addressing demographic (e.g., age, race, gender) and sociographic (e.g., income, level of education, and occupation) variables. Specifically, the data were extracted for the inclusion of the 11 demographic domains (annual family income, birthplace, current address, current age, current employment status, educational attainment – individual, ethnicity and race, gender identity, health insurance coverage, sex assigned at birth, and sexual orientation), and five sociographic domains (access to health services, English proficiency, food insecurity, health literacy, occupational prestige) of the PhenX SDoH Core collection. Final extraction data items included review paper ID, authors, year, DOI, title, abstract, and SDoH-related variables (see Appendix 3). All eligible studies were included in the scoping review. The critical appraisal of evidence was not conducted due to the scoping nature of the systematic review reported in this paper.

### Statistical analyses

Descriptive statistics were used to report the total count and percent of paper papers reporting individual SDoH variables and means and standard deviations of the unique SDoH domains reported per paper. Linear regression was used to assess the trend and projection in SDoH reporting over years. Descriptive analyses were conducted in Excel, and SAS 9.4 software was used for the regression analysis.

## Results

A total of 837 papers published in the JCTS were identified and screened. After the title and abstract review, 428 were excluded, and 280 articles were excluded during the full-text review. After the screening, 129 papers were deemed eligible for inclusion. Included papers were published in 2017 (*n* = 5), 2018 (*n* = 10), 2019 (*n* = 9), 2020 (*n* = 23), 2021 (*n* = 18), 2022 (*n* = 20), and 2023 (*n* = 44). The complete list of included studies is available in Appendix 3.

### SDoH variables reported in JCTS papers in 2017–2023

Each individual and social domain covered by the PhenX SDoH Core collection was included in at least one study, and 118 (91.5%) studies reported at least one SDoH domain. Age (*n* = 99, 76.7%) and ethnicity and race (*n* = 98, 76.6%) were among the most frequently reported individual-level SDoH variables. Values for the sex assigned at birth variable are operationalized by the PhenX Core protocol as female, male, and intersex; 91 (70.5%) papers reported sex assigned at birth values but referred to them as gender. A few papers (*n* = 8, 6.2%) differentiated between sex and gender variables; the latter were reported using man, woman, non-binary, and transgender values. Other individual SDoH variables were reported for educational attainment (*n* = 49, 38.0%), annual family income (*n* = 20, 15.5%), health insurance coverage (*n* = 19, 14.7%), current employment status (*n* = 13, 10.1%), current address (*n* = 12, 9.3%), birthplace (*n* = 2, 1.6%), and sexual orientation (*n* = 2, 1.6%). Furthermore, SDoH variables other than age, ethnicity and race, and sex assigned at birth were reported in 72 (55.8%) papers.

**Figure 1.**
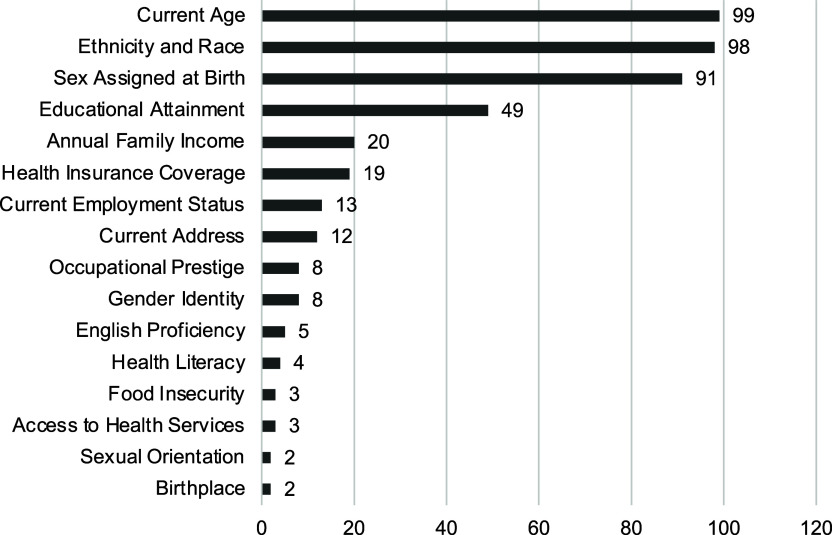
Number of Journal of Clinical and Translational Science papers reporting individual social determinants of health domains.

Sociographic SDoH variables were included to a much lesser degree: Access to health services (*n* = 3, 2.3%), English/language proficiency (*n* = 5, 3.9%), food insecurity (*n* = 3, 2.3%), health literacy (*n* = 4, 3.1%), and occupational prestige (8, 6.2%). Figure 1 shows graphically the number of JCTS papers that report on the PhenX SDoH domains.

### Annual trends in SDoH reporting

**Figure 2.**
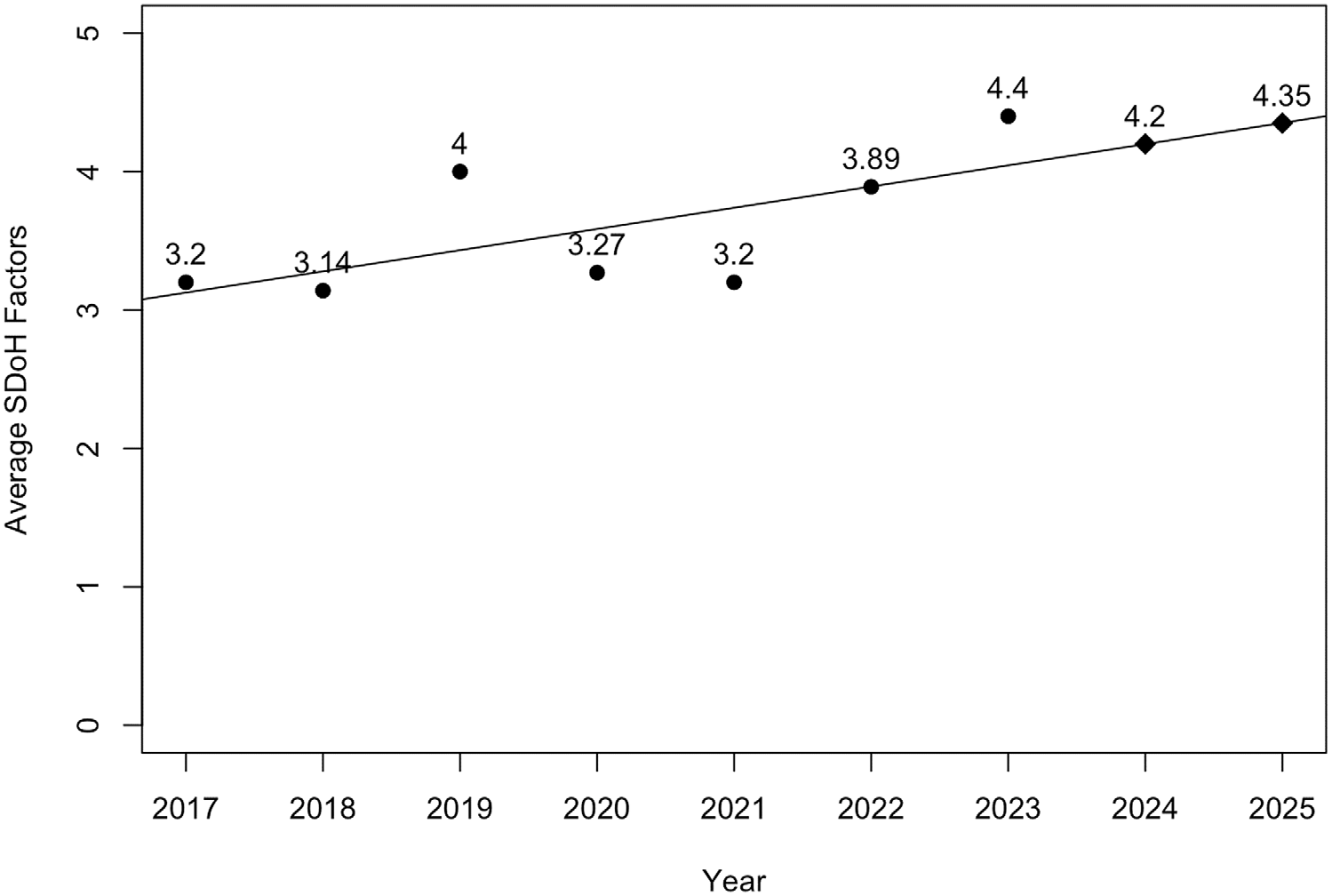
Linear regression with predictions of average social determinants of health (SDoH) factors per paper reported and projected in 2017–2025.

The average number of SDoH factors included in the JCTS empirical papers between 2017 and 2023 was 3.4 (*SD* = 1.78, Min = 0, Max = 8). Annual trends included inconsistent but discernable growth: 2017 (*M* = 3.20, *SD* = 0.83), 2018 (*M* = 2.40, *SD* = 1.89), 2019 (*M* = 3.55, SD = 1.81), 2020 (*M* = 2,56, *SD* = 1.80), 2021 (*M* = 3.33, *SD* = 1.32), 2022 (*M* = 3.75, *SD* = 1.77), and 2023 (*M* = 3.93, *SD* = 1.82). Figure 2 shows a linear regression analysis of the average number of SDoH factors reported per paper from 2017 to 2023, with predictions for 2024 and 2025. The linear regression line indicates a steady increase and forecasts an average of 4.20 SDoH factors for 2024 and 4.35 for 2025. The model predicts an increase to an average of 5 SDoH factors reported per paper, which is a small increase compared to the 2023 average.

The plotted data also show an increase in SDoH reporting in 2019, a decrease in 2020–2021, and new growth starting in 2022. We looked at the titles published in 2019–2021. None of the papers published in 2019 were related to COVID. These papers were likely conceptualized pre-COVID, considering the time it takes to collect and analyze data and see a paper through the review and publication process. Several papers in 2020 and 2021 report on COVID-related studies or acknowledge its effect. The qualitative review and the data trend suggest that COVID-19 had a negative effect on the SDoH reporting.

## Discussion

This paper reported a scoping review of empirical studies published in the *Journal of Clinical and Translational Science* between 2017 and 2023. This review was guided by the set of SDoH variables included in the PhenX SDoH Core collection. The use of standardized SDoH data capture protocols, as outlined in the PhenX Toolkit, has significant implications for clinical research. These methods enable researchers to consistently collect data, facilitating better comparison and integration of studies. This enhances understanding of the socioeconomic drivers of health outcomes. Efforts to include individual and social SDoH measures are critical to the conduct of translational research and the advancement of translational science. Each SDoH Core collection variable was included in at least one paper, but the review also revealed low consistency in reporting all recommended variables. Furthermore, almost half of the papers (45%) reported only the top three most common variables: age, race/ethnicity, and sex assigned at birth. Finally, the data suggest that COVID-19 may have had a negative impact on the capture and reporting of SDoH variables. This observation is limited to the papers published in the JCTS but warrants exploration as the effects of COVID-19 on the clinical and translational research processes, and the quality of data has been considered in previous research [23].

While limited to the papers published in the JCTS, this review has several important implications for translational research and science. First, most papers labeled sex assigned at birth variables as gender. The distinction between biological sex and socially constructed gender has been expensively explicated and discussed in academic literature. However, reporting these two constructs still needs more consistency and standardization. Investigators engaged in empirical clinical and translational research should use appropriate terminology when reporting study results. Future translational science studies are warranted and can explore the mental models, perceptions, and practices contributing to incorrect sex and gender terminology [24].

Second, this review showed that a minority of papers reported capturing the current address of study participants. This data may have been available as it is customarily collected in clinical and translational research but not considered for inclusion in the published papers. ZIP- or neighborhood-level data can be linked to the established social vulnerability indices and provide information about social determinants affecting research participants without subjecting them to additional data collection [25]. In addition, the use of individually collected demographic and sociographic data and publicly available neighborhood-level data can inform the development of research participant phenotypes to inform the design and evaluation of recruitment and retention processes and research study outcomes [26,27].

Finally, this review showed that at least one study reported each SDoH construct suggested by the PhenX Core collection. At the same time, the range of reported SDoH constructs varied from zero to eight, and it is unclear if the variables were omitted due to oversights or because they were considered non-essential for the aims and designs of the individual studies. This signals an opportunity for point-of-care research to assess the participant and researcher burden, the feasibility, and the appropriateness of collecting the full core set of SDoH variables for research studies. To make the decisions and availability of the recorded SDoH data explicit, future studies can include a checklist stating the study design and data capture decisions. A draft checklist is provided in Appendix 4. However, additional collaborative, participatory efforts are needed to finalize the list, assess the feasibility of its use, conduct content validity studies, and develop consensus-based recommendations for its use.

## Conclusion

This systematic scoping review study found that from the first volume published in 2017 to December 2023, the number of SDoH variables reported in the JCTS empirical papers remained relatively low, with an upward trend for reporting individual and social participant data. Individual-level demographic variables accounted for most reported SDoH data, with age, race/ethnicity, and sex being the most reported variables. Sociographic variables were present in papers but reported at a much lower rate than individual-level variables, which presents an opportunity for research process improvement.

## Supporting information

Levites Strekalova et al. supplementary material Levites Strekalova et al. supplementary material

## Supplementary material

The supplementary material for this article can be found at https://doi.org/10.1017/cts.2024.508.
